# Evolutionary Recycling of Light Signaling Components in Fleshy Fruits: New Insights on the Role of Pigments to Monitor Ripening

**DOI:** 10.3389/fpls.2016.00263

**Published:** 2016-03-07

**Authors:** Briardo Llorente, Lucio D’Andrea, Manuel Rodríguez-Concepción

**Affiliations:** Centre for Research in Agricultural Genomics (CRAG) CSIC-IRTA-UAB-UB Barcelona, Spain

**Keywords:** photosensory pathways, light, fleshy fruits, ripening, evolution

## Abstract

Besides an essential source of energy, light provides environmental information to plants. Photosensory pathways are thought to have occurred early in plant evolution, probably at the time of the Archaeplastida ancestor, or perhaps even earlier. Manipulation of individual components of light perception and signaling networks in tomato (*Solanum lycopersicum*) affects the metabolism of ripening fruit at several levels. Most strikingly, recent experiments have shown that some of the molecular mechanisms originally devoted to sense and respond to environmental light cues have been re-adapted during evolution to provide plants with useful information on fruit ripening progression. In particular, the presence of chlorophylls in green fruit can strongly influence the spectral composition of the light filtered through the fruit pericarp. The concomitant changes in light quality can be perceived and transduced by phytochromes (PHYs) and PHY-interacting factors, respectively, to regulate gene expression and in turn modulate the production of carotenoids, a family of metabolites that are relevant for the final pigmentation of ripe fruits. We raise the hypothesis that the evolutionary recycling of light-signaling components to finely adjust pigmentation to the actual ripening stage of the fruit may have represented a selective advantage for primeval fleshy-fruited plants even before the extinction of dinosaurs.

## Introduction

Light has a dual role in plants as an essential source of energy for driving photosynthesis and, on the other hand, as an environmental cue that modulates many aspects of plant biology such as photomorphogenesis, germination, phototropism, and entrainment of circadian rhythms (Chen et al., 2004; Jiao et al., 2007). The ability to perceive and respond to light changes is mediated by a set of sophisticated photosensory pathways capable of discriminating the quality (spectral composition), intensity (irradiance), duration (including day length), and direction of light (Moglich et al., 2010). In particular, plants perceive light through at least five types of sensory photoreceptors that are distinct from photosynthetic components and detect specific regions of the electromagnetic spectrum. Cryptochromes (CRYs), phototropins, and Zeitlupe family members function in the blue (390–500 nm) and ultraviolet-A (320–390 nm) wavelengths, while the photoreceptor UVR-8 operates in the ultraviolet-B (280–315 nm) region. Phytochromes (PHYs), which are probably the best studied photoreceptors, function in a dynamic photoequilibrium determined by the red (R, ca. 660 nm) to far-red (FR, ca. 730 nm) ratio in land plants and throughout the visible spectrum (blue, green, orange, red, and far-red) in different algae (Moglich et al., 2010; Rizzini et al., 2011; Rockwell et al., 2014). The photonic information gathered by these photoreceptors is then transduced into changes in gene expression that ultimately promote optimal growth, development, survival and reproduction (Jiao et al., 2007).

Photosensory pathways are thought to have occurred early in plant evolution, probably at the time of the Archaeplastida ancestor (i.e., the last common ancestor of glaucophyte, red algae, green algae and land plants) or perhaps even earlier, before the occurrence of the endosymbiotic event that gave rise to photosynthetic eukaryotes over more than a billion years ago (Duanmu et al., 2014; Mathews, 2014; Fortunato et al., 2015). Through the ages, these mechanisms diverged to play particular roles in different branches of the plant lineage, ranging from presumably acclimative roles in algae (Duanmu et al., 2014; Rockwell et al., 2014) to resource competition functions in land plants (Jiao et al., 2007). In particular, the ability of PHYs to detect changes in the R/FR ratio allows land plants to detect the presence of nearby vegetation that could potentially compete for light. Light filtered or reflected by neighboring leaves (i.e., shade) has a distinctive spectral composition that is characterized by a decreased R/FR ratio due to a preferential absorption of R light by chlorophyll (Casal, 2013). Low R/FR ratios reduce PHY activity, allowing PHY-interacting transcription factors (PIFs) to bind to genomic regulatory elements that tune the expression of numerous genes (Casal, 2013; Leivar and Monte, 2014). Oppositely, high R/FR ratios enhance PHY activity, causing the inactivation of PIF proteins mainly by proteasome-mediated degradation (Bae and Choi, 2008; Leivar and Monte, 2014). Carotenoid biosynthesis represents a rather well characterized example of this regulation. In *Arabidopsis thaliana*, shade decreases the production of carotenoids in photosynthetic tissues (Roig-Villanova et al., 2007; Bou-Torrent et al., 2015) in part by promoting the accumulation of PIF proteins that repress the expression of the gene encoding phytoene synthase (PSY), the main rate-determining enzyme of the carotenoid pathway (Roig-Villanova et al., 2007; Toledo-Ortiz et al., 2010; Bou-Torrent et al., 2015). De-repression of *PSY* under sunlight induces carotenoid biosynthesis, which in turn maximizes light harvesting and protects the photosynthetic machinery from harmful oxidative photodamage caused by intense light (Sundstrom, 2008).

Light signals in general and PHYs in particular also modulate the genetic programs associated to fruit development and ripening. Here we will revise current and emerging knowledge on this area based on work carried out in tomato (*Solanum lycopersicum*), which is the main model system for fleshy fruits, that is, fruits containing a juicy fruit pulp. Further, we will discuss potential selection pressures that might account for the evolutionary recycling of light-signaling components in fleshy fruits.

## Fleshy Fruit Ripening: The Case of Tomato

Fleshy fruits are differentiated floral tissues that evolved 80–90 million years ago (Ma), i.e., relatively recently in the history of plants (Givnish et al., 2005; Eriksson, 2014), as an adaptive characteristic promoting the animal-assisted dissemination of viable seeds (Tiffney, 2004; Seymour et al., 2013; Duan et al., 2014). After seed maturation, fleshy fruits typically undergo a ripening process that involves irreversible changes in organoleptic characteristics such as color, texture, and flavor, all of which result in the production of an appealing food to frugivorous animals. In this manner, the ripening process orchestrates the mutualistic relationship between fleshy-fruited plants and seed-disperser animals (Tiffney, 2004; Seymour et al., 2013; Duan et al., 2014).

Upon fertilization, the development of fleshy fruits such as tomato can be divided into three distinct phases: cell division, cell expansion, and ripening (Gillaspy et al., 1993; Seymour et al., 2013). These different stages are characterized by hormonal, genetic, and metabolic shifts that have been reviewed in great detail elsewhere (Carrari and Fernie, 2006; Klee and Giovannoni, 2011; Seymour et al., 2013; Tohge et al., 2014). Before ripening occurs, tomato fruits have a green appearance due to the presence of chloroplasts that contain the whole photosynthetic machinery. The transition to ripening is characterized by a loss of chlorophylls, cell wall softening, accumulation of sugars, and drastic alterations in the profile of volatiles and pigments. Most distinctly, chlorophyll degradation is accompanied by a conversion of chloroplasts into chromoplasts that progressively accumulate high levels of the health-promoting carotenoids β-carotene (pro-vitamin A) and lycopene (Tomato Genome Consortium, 2012; Fantini et al., 2013; Seymour et al., 2013). These carotenoid pigments give the characteristic orange and red colors to ripe tomatoes. A large number of other fruits (including bananas, oranges, or peppers) also lose chlorophylls and accumulate carotenoids during ripening, resulting in a characteristic pigmentation change (from green to yellow, orange or red) that acts as a visual signal informing animals when the fruit is ripe and healthy (Klee and Giovannoni, 2011).

## The Effect of Light Signaling Components on Fruit Ripening

**FIGURE 1:**
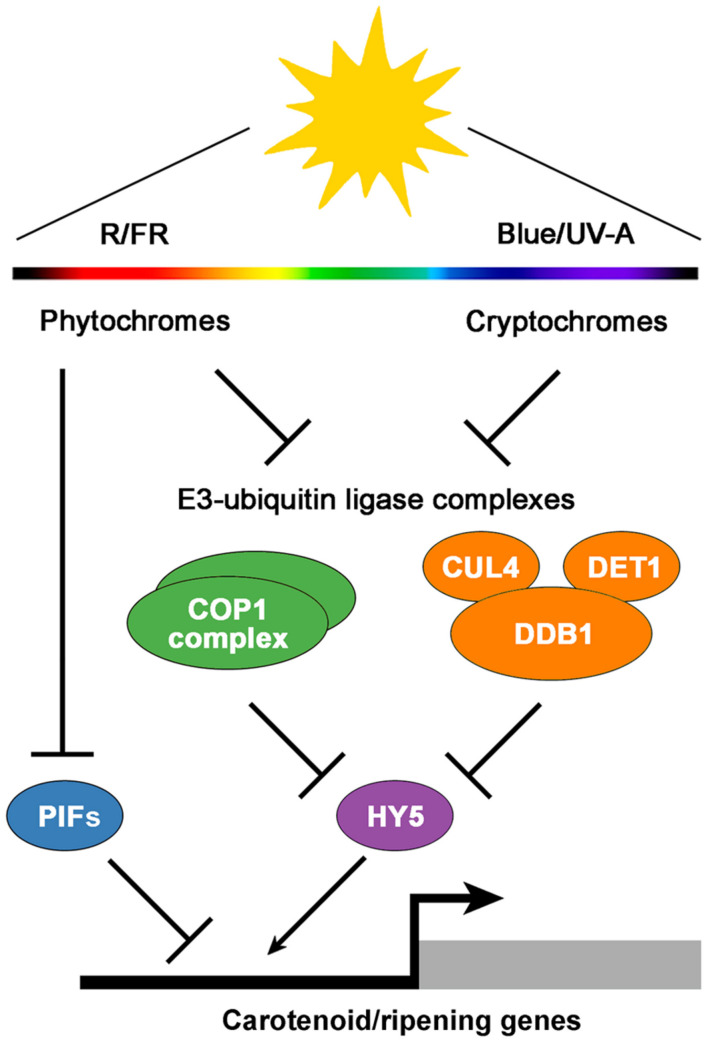
**A simplified model of light signaling components involved in the regulation of tomato fruit pigmentation and ripening.** Fruit-localized phytochrome and cryptochrome photoreceptors regulate the activity of the downstream E3-ubiquitin ligase COP1 and CUL4-DDB1-DET1 complexes, which in turn mediate the degradation of the transcriptional activator HY5. In addition, active phytochromes reduce the activity of transcriptional repressors such as PIFs. The balance between activators and repressors finally modulates the expression of carotenoid and ripening-associated genes. R, red light; FR, far-red light; Blue, blue light; UV-A, ultraviolet-A light.

Multiple lines of evidence have exposed the relevance of fruit-localized photosensory pathways as important players in the regulation of fruit ripening and the potential of their manipulation to improve the nutritional quality of tomatoes (Azari et al., 2010). Among many light-signaling mutants displaying altered fruit phenotypes, the tomato high pigment (*hp*) mutants *hp1* and *hp2* are two of the best characterized. These mutants owe their name to a deep fruit pigmentation derived from an increment in the number and size of plastids, which in turn result in elevated levels of carotenoids such as lycopene (Yen et al., 1997; Mustilli et al., 1999; Levin et al., 2003). Detailed characterization of the *hp1* and *hp2* mutants, which also show increased levels of extraplastidial metabolites such as flavonoids, revealed that the mutated genes encode tomato homologs of the previously described light signal transduction proteins DAMAGED DNA BINDING PROTEIN 1 (DDB1) and DEETIOLATED1 (DET1), respectively (Mustilli et al., 1999; Schroeder et al., 2002; Levin et al., 2003; Liu et al., 2004) (**Figure 1**). Other components that participate in the same light-signaling pathway that HP1 and HP2 have also been shown to impact tomato fruit metabolism. For instance, silencing the tomato E3 ubiquitin-ligase CUL4, which directly interacts with HP1, also produces highly pigmented fruits (Wang et al., 2008). Another example is the E3 ubiquitin-ligase CONSTITUTIVELY PHOTOMORPHOGENIC 1 (COP1), which specifically promotes the degradation of the light-signaling effector ELONGATED HYPOCOTYL 5 (HY5) (Schwechheimer and Deng, 2000) (**Figure 1**). Transgenic plants with downregulated transcripts of COP1 and HY5 produce tomato fruits with increased and reduced levels of carotenoids, respectively (Liu et al., 2004).

Work with photoreceptors (**Figure 1**) has also shed light on the subject. Tomato plants overexpressing the blue light photoreceptor cryptochrome 2 (CRY2) produce fruits with increased levels of flavonoids and carotenoids (Giliberto et al., 2005). PHYs have been found to control different aspects of tomato fruit ripening as well. Activation of fruit-localized PHYs with R light treatments promotes carotenoid biosynthesis, while subsequent PHY inactivation by irradiation with FR light reverts it (Alba et al., 2000; Schofield and Paliyath, 2005). Furthermore, preventing light exposure from the very early stages of fruit set and development results in white fruits completely devoid of pigments (Cheung et al., 1993), a phenotype that resembles that of *phyA phyB1 phyB2* PHY triple mutant plants (Weller et al., 2000). In addition to regulating carotenoid levels in tomato fruits, PHYs seem to regulate the timing of phase transition during ripening (Gupta et al., 2014).

## A Mechanism to Monitor Ripening Based on Self-Shading and Light Signaling

**FIGURE 2:**
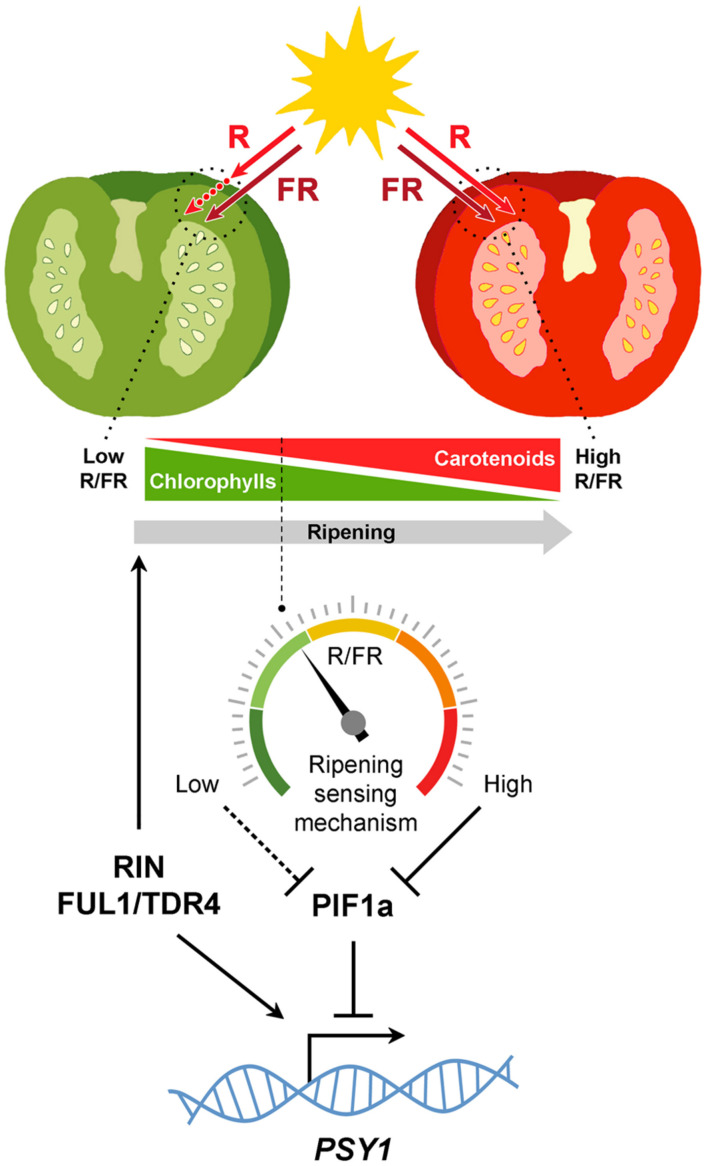
**Self-shading model for the light mediated modulation of carotenoid biosynthesis in tomato fruits.** Chlorophylls in green fruits preferentially absorb red (R, ca. 660 nm) wavelengths of the light spectrum, generating a self-shading effect characterized by low R to far-red (FR, ca. 730 nm) ratios that maintain PHYs predominantly in the inactive form and relatively high levels of PIF1a repressing *PSY1*. Once seeds mature, the developmental program induces the expression of genes encoding master activators of the ripening process. Some of them, like RIN and FUL1/TDR4, also induce *PSY1* gene expression directly. Chlorophyll breakdown reduces the self-shading effect so that the R/FR ratio within the cells gradually increase, consequently displacing PHYs to their active form, reducing PIF1a levels and derepressing *PSY1* expression. By sensing the spectral composition of the light filtered through the fruit pericarp, this mechanism diagnoses actual ripening progression to finely adjust fruit carotenoid biosynthesis.

Although light signaling components have long been known to modulate fruit ripening, another important piece of the puzzle was revealed recently. In tomato, fruit pericarp cells are morphologically similar to leaf palisade cells (Gillaspy et al., 1993). Thus, fruits can be viewed as modified leaves that, besides enclosing the seeds, have suffered a change in organ geometry, namely, a shift from a nearly planate conformation to an expanded three-dimensional anatomy. This anatomy imposes spatial constrains coercing light to pass through successive cell layers, so that the quality of the light that reaches inner sections of the fruit is influenced by the cells of outer pericarp sections (**Figure 2**). Another key difference between tomato leaves and fruits is the cuticle, which is far more pronounced in the fruit. While a potential role of the cuticle in altering the spectral properties of the light that reaches the pericarp cells remains to be investigated, it is now well established that the occurrence of chlorophyll in fruit chloroplasts significantly reduces the R/FR ratio of the light filtered through the fruit fresh (Alba et al., 2000; Llorente et al., 2015). A reduction in R/FR ratio (also referred to as shade) normally informs plants about the proximity of surrounding vegetation (Casal, 2013). In tomato fruit, however, changes in R/FR ratio can inform of the ripening status. As a consequence of self-shading, it is proposed that a relatively high proportion of PHYs remain inactive in green fruit. This condition stabilizes the tomato PIF1a transcription factor, that binds to a PBE-box located in the promoter of the gene encoding the PSY isoform that controls the metabolic flux to the carotenoid pathway during fruit ripening, *PSY1*. PIF1a binding directly represses *PSY1* expression (**Figure 2**). Chlorophyll breakdown at the onset of ripening reduces the self-shading effect, consequently promoting PHY activation, degradation of PIF1a, derepression of *PSY1*, and eventually carotenoid biosynthesis (**Figure 2**). In this manner, the genetically controlled expression of *PSY1* (and hence the production of carotenoid pigments) is fine-tuned to the actual progression of ripening (Llorente et al., 2015).

Translation of molecular insights from tomato to other fleshy-fruited plants has indicated that many regulatory networks are conserved across a wide range of species (Seymour et al., 2013). Thus, given the ubiquitous nature of PHYs in land plants and the widespread occurrence of ripening-associated fruit pigmentation changes that typically involve the substitution of an initially chlorophyll-based green color with distinctive non-green (i.e., non-R-absorbing) eye-catching colors, it is possible that similar self-shading regulatory mechanisms might operate in other plant species to inform on the actual stage of ripening (based on the pigment profile of the fruit at every moment) and thus finely coordinate fruit color change. However, the composition of the cuticle or even the anatomy of the most external layer of the pericarp (i.e., the exocarp) might also impact the quality and quantity of light that penetrates the fruit flesh. The self-shading mechanism is expected to be irrelevant in fleshy fruits with a thick skin or exocarp that prevents light to pass through and reach more internal fruit layers.

## Fruit Colors as Ripening Signals in an Evolutionary Context

Fleshy fruits are considered to have first appeared in the Late Cretaceous (circa 90 Ma) (Givnish et al., 2005; Eriksson, 2014), at a time when the Earth’s vegetation was dense and exuberant, and where most ecological niches were taken over by angiosperms (Lidgard and Crane, 1988; Berendse and Scheffer, 2009). The plentiful surplus of nutritious food gave rise to a huge explosion in the Cretaceous fauna, bringing about the coexistence of numerous herbivorous and omnivorous reptiles (dinosaurs, pterosaurs, lizards), birds and mammals (Lloyd et al., 2008; Prentice et al., 2011; Vullo et al., 2012; Wilson et al., 2012; Jones et al., 2013; Jarvis et al., 2014). With such an abundance of plant-eating animals, being able to display a change in fruit color when ripe probably represented a valuable trait among early fleshy-fruited plants to call the attention of these various potential seed dispersers.

Although deep time co-evolutionary scenarios may be difficult to support, this idea gains plausibility if we consider that the same strategy had been successfully implemented beforehand by gymnosperms, which had already evolved fleshy fruit-like structures by the Early Cretaceous, at least some 20-30 million years before the first fleshy fruits (Yang and Wang, 2013). Several gymnosperms (e.g., *Ginkgo biloba*, *Taxus baccata*, and *Ephedra distachya*) produce fleshy colorful tissues around their seeds and, similar to that occurring in angiosperms, these fruit-like structures undergo a ripening process that also serves as a visual advertisement for animals to eat them and disperse their seeds. Recent evidence supports the hypothesis that the main molecular networks underlying the formation of the fleshy fruit were originally established in gymnosperms (Lovisetto et al., 2012, 2015), thus suggesting that the ripening phenomenon was first selected as an ecological adaptation in gymnosperms and that angiosperms merely exploited it afterwards. If correct, this would imply that Cretaceous plant-eater animals would have already been used to feeding on color-changing fleshy fruit-like tissues by the time that angiosperm fleshy-fruited plants evolved, something that may have facilitated the establishment of the latter.

Another relevant fact is that the dominant land animals during the Cretaceous period, the dinosaurs, as well as pterosaurs, lizards, and birds, had highly differentiated color vision, much superior to that of most mammals (Rowe, 2000; Chang et al., 2002; Bowmaker, 2008). Differentiated color vision, or tetrachromacy, is a basal characteristic of land vertebrates derived from the presence of four spectrally distinct retinal cone cells that allow discriminating hues ranging from ultraviolet to red (Bowmaker, 2008; Koschowitz et al., 2014). Turtles, alligators, lizards and birds, are all known to have tetrachromatic color vision, a shared trait inherited from their common reptilian ancestry (Rowe, 2000; Bowmaker, 2008). We have recently come to know that some dinosaurs even sported plumage color patterns and flamboyant cranial crests that may have served for visual display purposes (Li et al., 2010, 2012; Zhang et al., 2010; Bell et al., 2014; Foth et al., 2014; Koschowitz et al., 2014). Altogether, these insights suggest that color cues were likely an important means of signaling among dinosaurs. Although purely speculative at the moment, it is reasonable to assume that there could have also been dinosaurs that, analogously to several birds and reptiles nowadays (Svensson and Wong, 2011), consumed fleshy fruits within their diet as a source of carotenoid pigments used for ornamental coloration. Even though the relevance of, now extinct, Cretaceous megafauna as biological vectors involved in the seed dispersal of primeval fleshy-fruited plants remains speculative and controversial (Tiffney, 2004; Butler et al., 2009; Seymour et al., 2013), it is clear that they certainly had fleshy fruit available to eat during the last 25–35 million years of their existence, until the occurrence of the Cretaceous-Paleogene mass extinction event (65 Ma).

Fruit color change meets the criteria of a classical signal, which can be defined as a cue that increases the fitness of the sender (i.e., fleshy-fruited plants) by altering the behavior of the receivers (i.e., seed-disperser animals) (Maynard Smith and Harper, 1995). Importantly, besides visibility conditions and the visual aptitude of the receiver, the detectability of a visual signal is determined by its contrast against the background, that is, the conspicuousness of the signal (Schmidt et al., 2004). Ripe fruits displaying a distinct coloration against the foliage leaves are more conspicuous for animals than green fruits and there is no evidence to consider that it was any different to Cretaceous animals. In fact, the invention of fruit fleshiness took place along with expanding tropical forests, suggesting it may have evolved as an advantageous trait related to changes in vegetation from open to more closed environments (Seymour et al., 2013; Eriksson, 2014). In this context, light signaling pathways already established in land plants may have had the chance to evolutionary explore novel phenotypic space in fleshy fruits. Subsequent adaptations under selection in the fruit may have then integrated these pathways as modulatory components of the pigmentation process during ripening. For instance, the self-shading regulation of the tomato fruit carotenoid pathway (Llorente et al., 2015) (**Figure 2**) might have evolved by co-option of components from the preexisting shade-avoidance responses (Mathews, 2006; Casal, 2013). This evolutionary recycling of light-signaling components in fleshy fruits might therefore be a legacy from the time when dinosaurs walked the earth.

## Author Contributions

BL, LA, and MR-C searched and discussed the literature and wrote the article.

## Conflict of Interest Statement

The authors declare that the research was conducted in the absence of any commercial or financial relationships that could be construed as a potential conflict of interest.
