# Corrigendum: Evolutionary Recycling of Light Signaling Components in Fleshy Fruits: New Insights on the Role of Pigments to Monitor Ripening

**DOI:** 10.3389/fpls.2022.900067

**Published:** 2022-04-05

**Authors:** Briardo Llorente, Lucio D'Andrea, Manuel Rodríguez-Concepción

**Affiliations:** Centre for Research in Agricultural Genomics (CRAG) CSIC-IRTA-UAB-UB, Barcelona, Spain

**Keywords:** photosensory pathways, light, fleshy fruits, ripening, evolution

In the original article, we neglected to include some funder information. The corrected ACKNOWLEDGMENTS section should be the following:

The authors apologize for this error and state that this does not change the scientific conclusions of the article in any way. The original article has been updated.

## Publisher's Note

All claims expressed in this article are solely those of the authors and do not necessarily represent those of their affiliated organizations, or those of the publisher, the editors and the reviewers. Any product that may be evaluated in this article, or claim that may be made by its manufacturer, is not guaranteed or endorsed by the publisher.

